# Spatial Variation in Background Mortality among Dominant Coral Taxa on Australia's Great Barrier Reef

**DOI:** 10.1371/journal.pone.0100969

**Published:** 2014-06-24

**Authors:** Chiara Pisapia, Morgan S. Pratchett

**Affiliations:** 1 ARC Centre of Excellence for Coral Reef Studies, James Cook University, Townsville, Queensland, Australia; AIMS@JCU, Australian Institute of Marine Science, School of Marine Biology, James Cook University, Townsville, Queensland, Australia; The Australian National University, Australia

## Abstract

Even in the absence of major disturbances (e.g., cyclones, bleaching), corals are consistently subject to high levels of background mortality, which undermines individual fitness and resilience of coral colonies. Partial mortality may impact coral response to climate change by reducing colony ability to recover between major acute stressors. This study quantified proportion of injured versus uninjured colonies (the prevalence of injuries) and instantaneous measures of areal extent of injuries across individual colonies (the severity of injuries), in four common coral species along the Great Barrier Reef in Australia: massive *Porites*, encrusting *Montipora*, *Acropora hyacinthus* and *Pocillopora damicornis*. A total of 2,276 adult colonies were surveyed three latitudinal sectors, nine reefs and 27 sites along 1000 km^2^ on the Great Barrier Reef. The prevalence of injuries was very high, especially for *Porites* spp (91%) and *Montipora* encrusting (85%) and varied significantly, but most lay at small spatial scales (e.g., among colonies positioned <10-m apart). Similarly, severity of background partial mortality was surprisingly high (between 5% and 21%) but varied greatly among colonies within the same site and habitat. This study suggests that intraspecific variation in partial mortality between adjacent colonies may be more important than variation between colonies in different latitudinal sectors or reefs. Differences in the prevalence and severity of background partial mortality have significant ramifications for coral capacity to cope with increasing acute disturbances, such as climate-induced coral bleaching. These data are important for understanding coral responses to increasing stressors, and in particular for predicting their capacity to recover between subsequent disturbances.

## Introduction

Disturbances play an important role in structuring natural communities [1], [2], especially in coral reef ecosystems, which are being subject to increasing frequency, severity and diversity of acute disturbances [1], [3]. Importantly, climate-related disturbances are compounding numerous pre-existing natural and anthropogenic disturbances [1], [4], [5], contributing to extensive coral loss and associated degradation of coral reef habitats [5], [6], [7], [8]. Coral reefs are highly dynamic ecosystems, naturally subject to a wide range of disturbances operating at different temporal and spatial scales, ranging from widespread mass bleaching events to chronic localised removal of live coral tissue by corallivores [9]. However, increasing effects of global climate change and other more direct anthropogenic disturbances appear to be increasing rates of coral mortality beyond those which can be sustained [10]. It is also possible that the capacity of corals to recover from successive disturbance events is declining, due to sustained declines in coral growth or reproductive output [8].

Coral assemblages can recover quite quickly in the aftermath of major disturbances (e.g., [11], [12]), as long as there is i) sufficient time between major disturbances, ii) adequate proximity or connectivity to viable source populations, and iii) maintenance of suitable substrates for settlement and subsequent survival of coral recruits [13]. Given increasing incidence of major disturbances (e.g., cyclones, bleaching or outbreaks of crown-of-thorns starfish), the interval between these events is often less than five years [14], exceeding the time needed for effective recovery [15]. Moreover, the increasing spatial extent of disturbances (e.g., climate-induced coral bleaching) is causing comprehensive mortality over very large areas, further undermining the capacity for recovery (but see [16]). The capacity for coral assemblages to recover can also be negatively affected by smothering or overgrowth by macroalgae, in instances where there is insufficient grazing by herbivorous fishes [17], and by a range of factors that potentially limit the early post-settlement growth and survivorship of corals [18].

While acute disturbances (e.g., cyclones, bleaching or outbreaks of crown-of-thorns starfish) often have very conspicuous effects on corals, causing high levels of whole colony mortality, chronic disturbances (e.g., predation, competition and disease) can have equally important effects on coral communities, further increase susceptibility to major disturbances [19] and greatly reduce recovery and resilience [20]. Wakeford and co-workers [20] attributed low coral cover recorded at Lizard Island, in northern Great Barrier Reef (GBR) to chronic disturbances and high rates of background mortality. Accordingly, coral assemblages at very isolated reefs (e.g., Scott Reef, off the north-west shelf), which are largely isolated from chronic anthropogenic disturbances, may recover quite rapidly even after very severe acute disturbances [16]. In the absence of major disturbances, corals are continually subject to a range of chronic, often small-scale disturbances that can cause relatively high rates of mortality [20], [21], [22], [23]. These chronic disturbances are a normal part of the natural dynamics and turnover in coral populations and communities [24], [25], [26], but may be increasing in prevalence and severity, thereby undermining the capacity for recovery (annual background mortality rates can generally vary from 1 to 30%: [20], [23], [26], [27], [28]. If for example, background mortality rates are increasing, the rate of recovery will be reduced, requiring an even longer period for complete recovery between successive major disturbances.

Corals are modular organisms and can survive extensive injury (loss of polyps) or partial mortality, on a scale far beyond the regenerative capacity of most solitary organism [29]. However, partial mortality and declines in the total number of polyps that make up a colony, result in smaller colony size, which can greatly affect individual fitness [24], . Colonies suffering from partial mortality must divert energy towards tissue repair, leading to a reduced energy expenditure towards growth, reproduction and other metabolic functions [32]. Very high prevalence and severity of partial mortality may therefore, have a stronger bearing on the fitness and fate of coral colonies and/or populations, than even colony size or other commonly used metrics of population structure. There are very few studies that have systematically quantified the prevalence or severity of partial mortality across a range of different corals or at a range of locations [33], [34]. It is very likely however, that rates of injury will vary spatially, with greatest variation likely to occur at relatively small scales [23]. On the GBR, for example, midshelf reefs are subject to frequent and severe acute disturbances, mostly associated with outbreaks of crown-of-thorns starfish [35], [36], whereas offshore reefs are relatively less affected by such disturbances. However, even more apparent is the patchy nature of most disturbances, such that some reefs may be severely impacted, whereas other nearby reefs are unaffected [3].

The aim of this study was to quantify the prevalence and severity of partial mortality across four dominant coral taxa (*Acropora hyacinthus*, *Pocillopora damicornis*, massive *Porites* spp and encrusting *Montipora*) at a hierarchy of spatial scales (among sectors, among reefs and among sites within reefs) on midshelf reefs on the Australia's GBR. Biotic and abiotic agents of partial mortality can vary in frequency, intensity and spatial scale and can therefore have different impacts on coral colonies at different scales [3]. By measuring the prevalence and severity of partial mortality across a hierarchy of different spatial scales we hope to provide insights into local versus global causes of partial mortality. Quantifying rates of tissue loss along the entire GBR is critical for understanding spatial variation in the recovery capacity and resilience of reef-building corals. More specifically, this study tests the hypothesis that background rates of partial mortality decrease with latitude, thereby accounting for apparent discrepancies in rates of population replenishment versus overall abundance of adult corals [37]. Similar levels of adult abundance despite much higher levels of recruitment in the northern GBR imply that there must be higher levels of background mortality. The greatest variation was expected between latitudinal sectors, however, since chronic background disturbances are recurrent patchy stressors, prevalence and severity of partial mortality were also expected to vary at smaller scale (reef and site). If so, then this may have significant ramifications for the capacity of corals to cope with increasing acute disturbances associated with global climate change.

## Materials and Methods

### Ethics statement

The activities for this study were conducted under permission from the Great Barrier Reef Marine Park Authority (Permit Number G09/32834.1). Only visual censuses of coral communities were conducted; no fauna or flora were collected or manipulated during this study.

### Study sites

**Figure 1:**
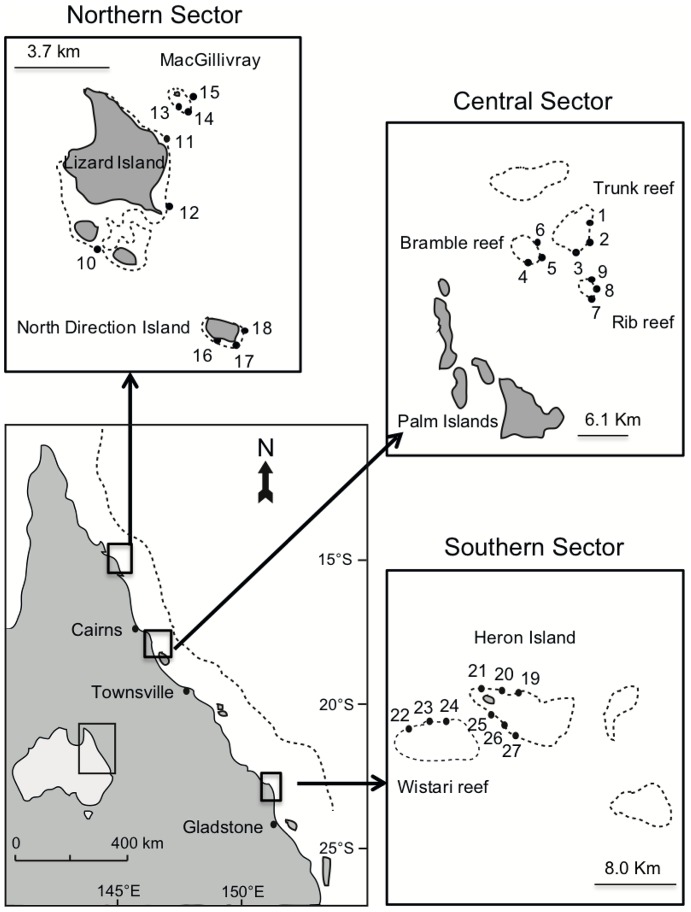
Map of the three study sectors (northern, central and southern) along the Great Barrier Reef, Australia. Within each latitudinal sector, three mid-shelf reefs, each with three sites on the exposed reef crest, were sampled. Figure adapted from Trapon and co-workers [18].

This study was conducted in 2011 on the Great Barrier Reef, Australia. Sampling was undertaken at a hierarchical of spatial scales. At the largest scale, three distinct latitudinal locations, separated by at least 500 km, were considered: the northern sector in the vicinity of Lizard Island (14°41′S, 145°28′E), the central sector, in vicinity of Trunk Reef (18 25′S, 146°47′E), and the southern sector, in the vicinity of Heron Island (23°27′S. 155°55′E) (Fig. 1). At each location, sampling was conducted at 2–3 reefs, and a total of nine different sites. Sites were separated by up to 2 km along the exposed (south-east) margin (Fig. 1). To avoid any effect of cross shelf variation, only mid-shelf reefs were sampled. Similarly, sampling was confined to reef crest habitats, between 3–5 m depth. At each site, sampling was conducted along three replicate 10×5 m belt transects orientated parallel to, and positioned within 5–10 m of, the reef crest. Due to the high frequency and intensity of disturbances (such as predation) on the reef crest, prevalence and severity of partial mortality were expected to be higher in this habitat compared to the flat and slope.

### Survey of partial mortality

This study considered four dominant coral taxa; *Acropora hyacinthus*, *Pocillopora damicornis*, massive *Porites* spp and encrusting *Montipora*. These four coral taxa are not only among the most common along the GBR [38], but represent contrasting morphologies and life-history strategies [39]. To compare the prevalence and severity of partial mortality within coral taxa, all relevant colonies located with the area of the each transect were surveyed and photographed. Each colony was photographed from the top with appropriate scale references, and all images were processed using the software Image J, to quantify the planar areal extent as a measure of colony size. The prevalence and severity of injuries were however, recorded *in situ* due to difficulties in capturing the full extent of injuries (especially on the sides and base of colonies) in a single photograph. The severity of partial mortality on individual colonies ranged from zero (no injury) to close to 100%. According to data from AIMS Long-term monitor program, during the study period the major acute disturbances that affected the three latitudinal sectors were cyclone Hamish in 2009 (southern GBR) and cyclone Yasi in 2011 (central and northern GBR). Since cyclones have a distinctive and identifiable effect on corals, such as dislodgment and breakage of colonies, it was easy to identify this agent of disturbance and exclude it from the survey. To reduce any bias broken or dislodged colonies were not included in the surveys, this ensured that partial mortality recorded during the survey was due to chronic rather than acute stressors.

### Data analysis

The effect of colony size on both prevalence and severity of tissue loss, regardless of spatial scales, was tested with two linear regressions for each species, using the proportion of injured colonies and arcsine partial mortality as dependent variables and colony size as predictor. Separate 1-way ANOVAs were then conducted for each of the four different taxa for both prevalence and severity of injuries. For prevalence of injuries, we analysed the proportion of colonies at each site that had any evidence of injury, and thereby compared prevalence of injury among sectors and among reefs (nested within sectors). For severity, mean colony percentage of partial mortality (which was arcsine transformed to meet the assumptions) was analysed among sectors, reefs, and sites (site nested within reef and reef nested within site). Variation in proportional mortality among size classes was analysed using size-frequency distributions, which were calculated based on estimates of the surface area of each colony. More specifically, size classes were determined *a priori* and were consistent across all coral taxa. The specific categories used correspond with log10 transformed surface area in cm^2^ of each colony following Bak and Meesters [40], and Adjeroud [41]. Spatial variation in both prevalence and severity of partial mortality (specifically, the proportional extent of dead versus living components of each colony) among the four coral taxa (*A. hyacinthus*, *P. damicornis*, massive *Porites* spp and encrusting *Montipora*) were analyzed using two separate hierarchically-nested ANOVAs, with transects (3 per site) nested within site, sites (3 per reef) nested within reef, and reefs (3 per sector) nested within latitudinal sectors (3 sectors). Proportional mortality for each colony was arcsine-square root transformed prior to analysis. Due to the unbalanced design, the F-statistic and p-values resulting from the Type III sum of square were reported. Variance components were also calculated for each coral taxa to assess whether the most important scale at which variation occurs is consistent across taxa with fundamentally different life-history dynamics. A Tukey's HSD post hoc test was used to identify where differences among group means occurred.

All tests were performed with STATISTICA 7.0 (StatSoft) software.

## Results

**Figure 2:**
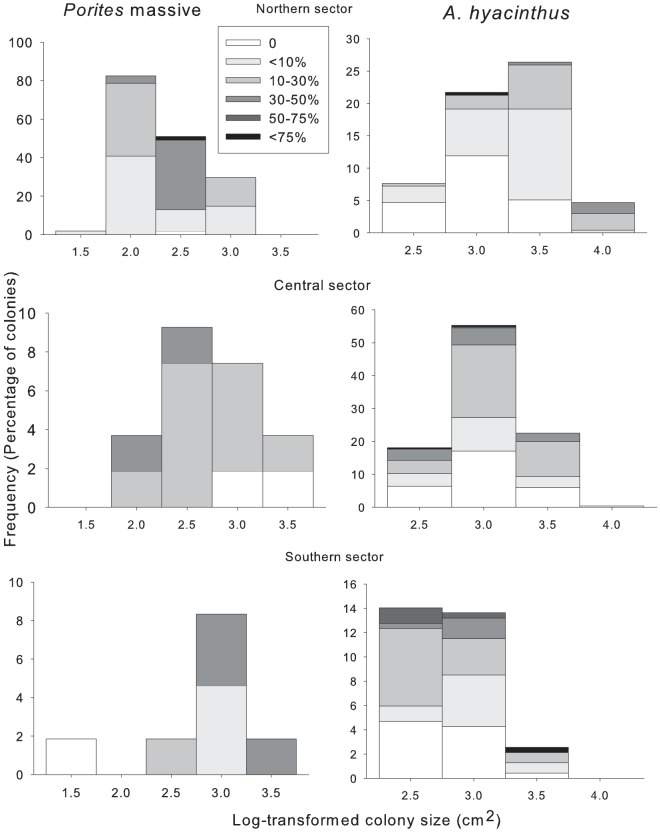
Size-frequency distributions (percentage of colonies) and percentage partial mortality for each size class in each latitudinal sector for *Porites* massive, and *Acropora hyacinthus*. Size classes were log10 transformed estimates of colony surface area.

**Figure 3:**
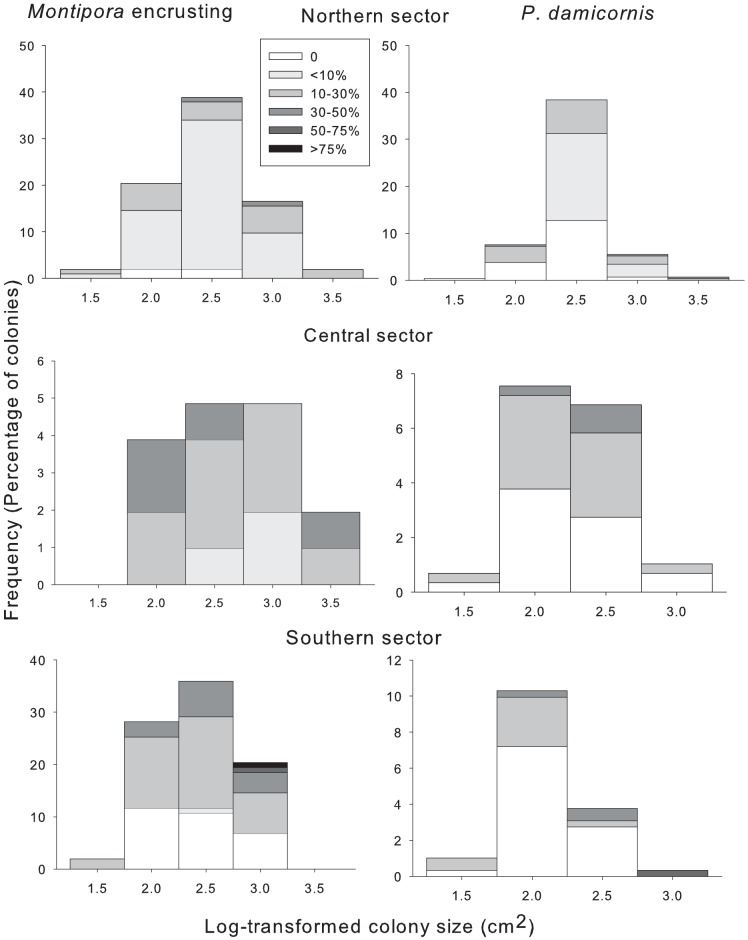
Size-frequency distributions (percentage of colonies) and percentage partial mortality for each size class in each latitudinal sector for encrusting *Montipora*, and *P. damicornis*. Size classes were log10 transformed estimates of colony surface area.

A total of 2,276 colonies were surveyed during this study, including 862 colonies of *A. hyacinthus*, 301 colonies of massive *Porites*, 505 colonies of *P. damicornis* and 608 colonies of encrusting *Montipora*. Prevalence of partial mortality or injuries was very high across all taxa, averaging 71% for *Acropora*, 59% for *P. damicornis*, 85% for encrusting *Montipora* and 92% for *Porites* across all sectors, reefs and sites (Table 1). All the four species exhibited a high range of tissue loss in all size classes: most size classes contained colonies with no mortality, moderate levels of mortality and extensive mortality (Fig. 2, 3). Also, the number of colonies with no injury, low, medium and high extent of injury was relatively similar among size classes (Fig. 2,3). This may explain why the regressions showed that both prevalence and severity of injury were generally independent of colony size in all the coral taxa (Table 2). Importantly, the r^2^ values were very low for all coral species indicating that colony size explained only a very small proportion of the total variation (Table 2). Colony size was a predictor of severity of partial mortality only in *P. damicornis* (r^2^ = 0.31, n = 294, p = 0.002), with smaller colonies having proportionately more injury (Table 2). The One-way ANOVAs results for tests of the regressions, investigating the relationship between prevalence of injury and severity of injury and colony size were not statistically significant in any of the coral taxa.

**Table 1:** Number of injured colonies versus number of total colonies surveyed at each site, within each reef and sector for the four coral species.

Sector	Reef	Site	*Acropora hyacinthus*	*Montipora* encrusting	*Porites* massive	*Pocillopora damicornis*
North	Lizard Island	1	9/16	56/56	16/16	10/13
North	Lizard Island	2	44/68	13/13	15/15	15/23
North	Lizard Island	3	0/0	14/14	41/43	12/24
North	Mac	1	9/14	6/6	28/28	21/38
North	Mac	2	34/50	9/9	12/12	3/4
North	Mac	3	30/60	37/37	17/17	20/34
North	North Direction	1	13/20	26/26	19/19	34/44
North	North Direction	2	16/19	40/42	32/32	35/62
North	North Direction	3	39/51	7/8	28/28	59/87
Central	Trunk	1	75/81	15/15	0/0	6/9
Central	Trunk	2	39/45	30/30	0/0	0/0
Central	Trunk	3	33/33	20/20	0/0	0/0
Central	Rib	1	60/60	3/3	15/15	10/10
Central	Rib	2	20/58	2/2	10/10	3/5
Central	Rib	3	12/40	9/9	10/10	2/3
Central	Bramble	1	19/21	10/10	9/15	2/3
Central	Bramble	2	35/60	32/32	19/19	8/20
Central	Bramble	3	21/23	10/10	0/0	6/11
South	Wistari	1	9/15	11/14	0/2	10/35
South	Wistari	2	12/18	8/13	2/2	2/3
South	Wistari	3	20/20	1/5	0/0	1/4
South	South Heron	1	11/24	9/15	10/10	20/20
South	South Heron	2	10/16	1/6	0/0	0/4
South	South Heron	3	27/37	33/66	0/0	2/21
South	North Heron	1	13/13	60/60	0/0	2/3
South	North Heron	2	0/0	17/27	5/5	25/25
South	North Heron	3	0/0	51/51	3/3	0/0

**Table 2:** Linear regression outcomes to investigate the effect of colony size on the severity (extent of injury) and on the prevalence (proportion of injured colonies) of partial mortality in each coral taxa.

Severity of partial mortality	R^2^	df	F	p
*A. hyacinthus*	0.007	1/468	3.7	0.05
*Porites* spp	0.05	1/109	3.25	0.59
*Montipora* encrusting	0.01	1/203	3.81	0.53
*P. damicornis*	0.31	1/292	9.56	0.002

Prevalence of partial mortality	R^2^	df	F	p
*A. hyacinthus*	0.49	1/22	1.14	0.29
*Porites* spp	0.01	1/17	0.01	0.89
*Montipora* encrusting	0.01	1/25	0.04	0.82
*P. damicornis*	0.09	1/22	0.2	0.65

Prevalence of partial mortality (proportion of injured colonies) varied significantly both among reefs and among sectors for *Montipora* encrusting (Table 3), with the greatest variation at sector level (54.1%). For all other coral taxa variation was most apparent at the smallest scales (within reefs), and there was no significant variation among reefs or among sectors.

**Table 3:** Results of hierarchically-nested ANOVAs to test for spatial variation in prevalence of injury (proportion of injured colonies per site) for each coral taxa.

	SS	df	MS	F	p	var (%)
*A. hyacinthus*						
Sector	0.07	2/16	0.03	0.89	0.42	0
Reef (Sector)	0.38	6/15	0.06	1.6	0.2	16.1
Error	0.6	16	0.04			83.9
*Porites* spp						
Sector	0.08	2/11	0.04	0.79	0.47	1.8
Reef (Sector)	0.34	5/11	0.06	1.31	0.32	1.2
Error	0.58	11	0.05			97
*Montipora* encrusting						
Sector	0.84	18/2	0.42	19.2	<0.001	54.1
Reef (Sector)	0.35	6/18	0.05	2.6	0.05	16.5
Error	0.39	18	0.02			29.4
*P. damicornis*						
Sector	0.06	2/15	0.03	0.51	0.6	0
Reef (Sector)	0.38	6/15	0.06	0.9	0.47	1.6
Error	0.1	15	0.7			98.4

**Figure 4:**
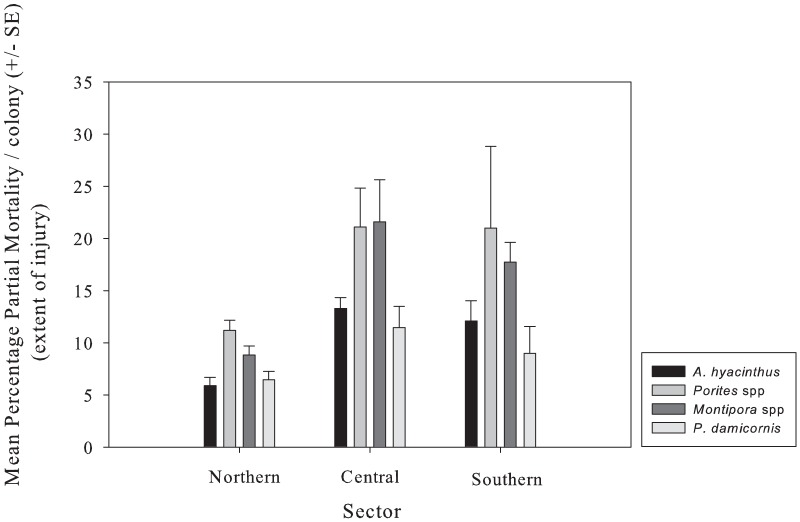
Mean (± SE) percentage partial mortality per colony (severity of partial mortality) in *Acropora hyacintus*, *Porites* massive, *Montipora* encrusting and *Pocillopora damicornis*, in the three latitudinal sectors: the northern, the central and the southern sector. Partial mortality was calculated as the proportion of dead to live tissue within the overall physical extent of each coral colony.

As for prevalence, the severity of partial mortality (the extent of injuries on individual colonies) varied most (>80%) at smallest scales, and in this case among transects within sites (Table 4). There was however, significant variation in the severity of partial mortality at the largest scale (among sectors) for *A. hyacinthus* and encrusting *Montipora*. For encrusting *Montipora*, mean severity of partial mortality was highest (21%) in the central sector, while in mean severity of partial mortality was highest (5%) for *A. hyacinthus* in the northern sector (Fig. 4).

**Table 4:** Results of hierarchically-nested ANOVAs to test for spatial variation in severity of partial mortality (specifically, the proportional extent of dead versus living components of each colony) for each coral taxa at all spatial scale.

	SS	df	MS	F	p	var (%)
*A. hyacinthus*						
Sector	1.15	2/450	0.57	13.15	>0.001	4.2
Reef (Sector)	0.58	4/450	0.14	3.32	0.01	14.2
Site (Sector*Reef)	0.5	4/450	0.12	2.87	0.02	0.4
Transect (Sector*Reef*Site)	0.13	4/450	0.03	0.76	0.54	0.2
Error	20.6	450	0.04			81
*Porites* spp						
Sector	0.03	2/94	0.01	0.67	0.51	8.3
Reef (Sector)	0.2	4/94	0.06	2.75	0.03	0
Site (Sector*Reef)	0.09	4/94	0.02	0.96	0.43	0
Transect (Sector*Reef*Site)	0.05	4/94	0.01	0.57	0.67	0.3
Error	2.27	94	0.02			91.4
*Montipora* encrusting						
Sector	0.38	2/185	0.19	4.8	>0.001	6.4
Reef (Sector)	0.27	4/185	0.06	1.7	0.1	4.2
Site (Sector*Reef)	0.07	4/185	0.01	0.45	0.7	4.9
Transect (Sector*Reef*Site)	0.06	4/185	0.01	0.38	0.8	1.1
Error	7.4	185	0.04			83.4
*P. damicornis*						
Sector	0.14	2/283	0.07	1.8	0.16	0.4
Reef (Sector)	0.14	4/283	0.03	0.93	0.44	1.4
Site (Sector*Reef)	0.25	4/283	0.06	1.61	0.17	1.4
Transect (Sector*Reef*Site)	0.2	4/283	0.05	1.29	0.27	5
Error	11.3	283	0.04			91.9

In *P. damicornis* and *Montipora* spp the majority of the colonies were smaller than 1000 cm^2^, while in *A. hyacinthus* colony surface area ranged from 90 cm^2^ up to 37.240 cm^2^, and in *Porites* ranged from 75 cm^2^ to 2410 cm^2^.

## Discussion

Prevalence of injury was consistent across all taxa and all locations, suggesting that background mortality is common along the GBR. Similarly, Wakeford and co-workers [19] found that annual background mortality at Lizard Island on the GBR, was ca 22% for *P. damicornis*, ca 18% for *A. hyacinthus* and ca 10% for *Porites* massive. This is much lower than recorded in this study, but Wakeford and co-workers [20] did not account for partial mortality. Even higher rates of annual background partial mortality were recorded in the Caribbean compared to the GBR. In Curacao in 2005 corals exhibited between 14 and 48% of tissue loss mainly due to disease [42], while in Bonairie in 2011 extent of injury ranged between 0 and 99% for *Montastrea* complex and was around 8% for other scleractinians [33]. Similarly, in Florida Keys, prevalence of recent partial mortality during periods of background, low-stress environmental conditions was still <5% for the 11 most abundant species [34]. High rates of background partial mortality within a population may lead to a decline in population densities through time because they can result in reduced colony growth [32], [43], reproductive output [44], [45], and reduced colony size [46] of individuals. Cumming [47] showed that recent injury can predict colony fate even more than colony size. However, even though partial mortality can negatively affect coral community dynamics [48], it also true that it may partially enable reef recovery by providing substrate for corals to settle, thus maintaining coral dominated reefs [33], [49].

The mean severity of background mortality varied among taxa suggesting that some species may be more resistant to routine agents and/or have better recovery potential. Massive *Porites* is a long-lived, slow growing coral, with generally low regenerative capabilities [50], [51], [52], [53] so old injuries are likely to accumulate through time. Conversely branching corals, such as *Acropora* have high regeneration capacities, rapid linear growth, and short generation time (less than 30 years) [41], [54]. The observed taxonomic differences in prevalence of injury recorded during this study broadly correspond with differences in growth rates and relative investment in repair [50], [51], [54], [55], [56], [57]. It is possible therefore, that *Acropora* have equal or higher incidence of injuries compared to massive *Porites*, but higher rates of whole colony mortality and/or more rapid regenerative capacity, leading to lower levels of instantaneous partial mortality. Branching corals, such as *Acropora* spp would intuitively appear much more vulnerable to breakage and injuries than massive corals [50]. However, the agents of partial mortality are likely to vary greatly among taxa. For example, fish predation is often not visible on branching corals, while it is conspicuous on massive *Porites*, which shows the highest rates of grazing scars compared to other coral species [58]. It appears however, that taxonomic differences in the severity of injury are most likely due to differences in persistence of injuries, or the rate of repair.

Previously published data documented marked latitudinal differences in population replenishment (highest in the northern GBR) despite similar adult abundance (measured as number of adult colonies per transect), suggesting that there are marked differences in the underlying dynamics of coral populations along the GBR [37]. More specifically, since high population replenishment did not correspond with high number of adult colonies, high rates of mortality (and/or reduced growth rates) may be responsible for differences between recruitment and adult populations. Based on these findings, it was expected background rates of mortality would be highest in the northern GBR and lowest in southern GBR. Conversely, background levels of partial mortality were found to be lower in the northern GBR and higher in the central and southern, with greatest variability apparent within sites or reefs, rather than among than sectors. Latitudinal gradients in key environmental variables (e.g., temperature and light) may in part influence rates of background mortality, modifying susceptibility or causing marked differences in recovery capacity. Temperature and light may affect background colony mortality by reducing or increasing regenerative abilities in colonies [53], [59], [60]. For instance reduced temperatures have been shown to cause polyps mortality [59] and declines in regeneration rates [53] and it may explain the higher rates of severity of partial mortality measured here in the southern GBR. Similarly, reduced light levels may cause a drop in regeneration rate due to reduced supply of photosynthetic products from zooxanthellae [53], [56], [60]. However, in the present study, latitude seemed to play a minor role in driving severity of background partial mortality, with only two of the four coral taxa showing latitudinal variation in the extent of injury.

Spatial variation in background mortality can greatly affect the response (e.g., capacity for recovery) of coral populations subject to increasing acute and anthropogenic disturbances. The drivers of the observed spatial variation are still unclear as the source of mortality was often hard to determine. Many *A. hyacinthus* and *P. damicornis* colonies had injuries at the edge of the colony suggesting that partial mortality was likely due to agents that were restricted to the bottom such as competition, polychaetes, or gravity causing scouring sand and moving coral fragments [50]. The reef-to-reef and site-to-site variability within sectors observed in *Porites* and *A. hyacinthus*, were likely the result of physical and biological routine agents such as fishes, echinoids, asteroids, molluscs, polychaetes, and microorganisms [61], [62], acting at spatial scales smaller, equal to or larger than individual reefs. These findings suggest that both disturbance regimes, and the responses of each species to routine agents are irregular, and may vary according to small differences in environmental conditions. The observed spatial variation in tissue loss supports results from other studies [20], [26], [63], [64], [65], [66], [67] showing how coral populations are subject to a wide range of different levels of disturbances and trajectories of recovery.

The most notable result from this study, is that variation in the prevalence and severity of partial mortality is most apparent at small (e.g., within reef) rather than larger, latitudinal scales. This shows that the disturbance history is likely to be more variable among colonies at the same site, than it is among disparate populations, suggesting that there is also likely to be marked variation in resilience to acute disturbances at this local scale [68], [69], [70]. Colonies with high incidence or severity of injuries are likely to have a generally lower capacity to withstand, and recover from, environmental changes or acute disturbances, leading to intraspecific differences in susceptibility to future acute disturbances. Accordingly, Pisapia et al [71] showed that adjacent colonies may vary greatly in their physiological condition due to localized differences in chronic disturbance regimes, though it is yet to be shown that this then leads to localized selectivity in the effects of major disturbances.

Mortality regimes of corals are expected to be strongly size-dependent, whereby the prevalence of partial mortality is expected to increase with colony size, while the probability of whole-colony mortality decreases with colony size [24], [46], [72], [73], [74], [75] because at least some portion of the colony is likely to persist in increasingly large colonies. In this study however, neither prevalence or severity of partial mortality showed a strong relationship with colony size; for *P. damicornis* there was a weak, though significant relationship between severity of injuries and colony size, but no such relationship existed for any other coral taxa. A lack of any relationship between size and severity of partial mortality was also observed in the Caribbean [33] where it was attributed to high variability in the extent of injury across all size classes. Moreover, differences in the repair and regenerative capacities of colonies of different sizes, may obscure such relationship, whereby larger colonies may experience higher incidence of injury, but also have greater capacity for tissue repair [24], [76], [77]. An injury of a given size is also going to require greater proportional investment in repair for smaller colonies [46], [78]. Therefore, repeated measurements through time of observable injuries on the same colonies are needed to better investigate recovery rates and capacity of these taxa.

Background levels of partial mortality are likely to have a fundamental effect on the fitness of individual colonies and the capacity of populations to withstand, and recover from, major acute disturbances [34], [48]. This is the first large-scale study of background levels of partial mortality, testing for large (latitudinal) and small (site) scale differences in the prevalence and severity of injuries across four dominant taxa of scleractinian corals. The findings from this study provide an insight in rates of mortality along the GBR, which can be used as a guideline for global comparisons and for evaluating environmental impacts on reefs or establishing monitoring projects. Instantaneous measures of observable injuries in adult coral colonies allow quantifying mortality events that are visible, and have lasting effects on coral colonies, as well as provide baseline estimates of coral mortality [48], [79]. However, since coral lesions regenerate at different rates, regeneration can stop before the injuries are fully healed or it can continue for over a year with lesions that do not initially regenerate healing later [80]. Future studies should combine instantaneous measures of observable injuries with repeated measurements over time to provide explicit estimates of the rate of injury. This research also needs to be combined with experimental studies to assess the effects of chronic injuries on colony physiological condition and on the capacity of colonies to withstand major acute disturbances
